# Intracellular Iron Chelation by a Novel Compound, C7, Reactivates Epstein–Barr Virus (EBV) Lytic Cycle via the ERK-Autophagy Axis in EBV-Positive Epithelial Cancers

**DOI:** 10.3390/cancers10120505

**Published:** 2018-12-11

**Authors:** Stephanie Pei Tung Yiu, Kwai Fung Hui, Chung King Choi, Richard Yi Tsun Kao, Chi Wang Ma, Dan Yang, Alan Kwok Shing Chiang

**Affiliations:** 1 Department of Paediatrics and Adolescent Medicine, Li Ka Shing Faculty of Medicine, The University of Hong Kong, Queen Mary Hospital, Pokfulam, Hong Kong SAR, China; stephanie.pty@gmail.com (S.P.T.Y.); kfhui@hku.hk (K.F.H.); miriamchoi10@gmail.com (C.K.C.); 2 Center for Nasopharyngeal Carcinoma Research, The University of Hong Kong, Hong Kong SAR, China; 3 Department of Microbiology, Li Ka Shing Faculty of Medicine, The University of Hong Kong, Hong Kong SAR, China; rytkao@hkucc.hku.hk; 4 Department of Chemistry, The University of Hong Kong, Hong Kong SAR, China; mcwhk@connect.hku.hk (C.W.M.); yangdan@hku.hk (D.Y.)

**Keywords:** iron chelation, Epstein–Barr virus, lytic cycle, gastric carcinoma, nasopharyngeal carcinoma, autophagy

## Abstract

Pharmaceutical reactivation of lytic cycle of Epstein–Barr virus (EBV) represents a potential therapeutic strategy against EBV-associated epithelial malignancies, e.g., gastric carcinoma (GC) and nasopharyngeal carcinoma (NPC). A novel lytic-inducing compound, C7, which exhibits structural similarity to Di-2-Pyridyl Ketone 4, 4-Dimethyl-3-Thiosemicarbazone (Dp44mT), a known chelator of intracellular iron, is found to reactivate EBV lytic cycle in GC and NPC. This study aims to investigate the role of intracellular iron chelation by C7 and other iron chelators in lytic reactivation of EBV in GC and NPC. Testing of six structural analogs of C7 revealed only those which have high affinity towards transition metals could induce EBV lytic cycle. Precomplexing C7 and iron chelators to iron prior to treatment of the cells abolished EBV lytic reactivation. Though hypoxia signaling pathway was activated, it was not the only pathway associated with EBV reactivation. Specifically, C7 and iron chelators initiated autophagy by activating extracellular signal-regulated kinase (ERK1/2) to reactivate EBV lytic cycle since autophagy and EBV lytic reactivation were abolished in cells treated with ERK1/2 blockers whilst inhibition of autophagy by 3-Methyladenine (3-MA) and *atg5* knockdown significantly abolished EBV lytic reactivation. In summary, we discovered a novel mechanism of reactivation of the EBV lytic cycle through intracellular iron chelation and induction of ERK-autophagy axis in EBV-positive epithelial malignancies, raising the question whether clinically available iron chelators can be incorporated into existing therapeutic regimens to treat these cancers.

## 1. Introduction

Epstein–Barr virus (EBV) is a ubiquitous γ-herpesvirus which infects more than 90% of the world population [1]. Its infection is generally asymptomatic if it occurs during childhood, yet the virus displays frequent association with various lymphomas and carcinomas. Of note, nasopharyngeal carcinomas (NPCs) in Southern China are constantly EBV-positive. EBV infection is also observed in a distinct subset of gastric carcinoma (GC). In these EBV-associated tumors, EBV persists in latent forms expressing a very restricted number of viral gene products. Upon lytic reactivation, a full spectrum of temporally-regulated viral lytic genes are expressed, which may then lead to cell death per se or target presentation for EBV-specific therapies [2]. One of the proposed EBV-targeted therapeutic strategies involves inducing the latent EBV in tumor cells into lytic cycle with pharmaceutical drugs. A viral protein kinase is being expressed, converting the co-administered antiviral drug, e.g., ganciclovir (GCV), from a prodrug to its cytotoxic form to kill the tumor cells [3]. Currently, various types of chemicals, including a number of histone deacetylase (HDAC) inhibitors [4,5], phorbol esters [6], and some chemotherapeutic drugs such as gemcitabine [7], have been shown to reactivate the EBV lytic cycle in vitro. However, the success of this strategy is limited by the refractoriness of cells to viral lytic cycle induction [8,9]. As reported in a study, the combinational use of gemcitabine, valproic acid (VPA), and GCV achieved partial response in two and stabilized diseases in three out of eight NPC patients [10]. To further develop this lytic induction strategy, identification of novel lytic inducers with potent and specific actions are required. 

A potent lytic cycle inducer, C7, was identified from a high-throughput screening in search of novel lytic inducers from a chemical library of 50,240 small synthetic organic compounds [11]. It contains a metal-binding moiety similar to iron chelators with thiosemicarbazone and aroylhydrazone structures. Iron chelators are currently used in the clinical setting mainly for removing excessive iron from the body in transfusion-dependent diseases [12]. Nevertheless, it has been shown that iron chelators also exhibit antitumor effects through targeting various survival pathways in cancer cells [13,14]. For instance, deferoxamine has been shown to inhibit TRAIL-mediated cancer cell apoptosis via autophagy induction in colon cancer cells [15]. Another study showed that deferasirox and deferoxamine induced autophagy and mediated cytotoxicity towards multiple myeloma [16]. Whist autophagy can play a role in protecting host cells from viral infection, viruses can also hijack autophagy proteins to promote their survival, replication, and exocytosis [17,18,19].

In this study, we aimed to investigate the role of intracellular iron chelation by C7 and different iron chelators, including Di-2-Pyridyl Ketone 4, 4-Dimethyl-3-Thiosemicarbazone (Dp44mT), deferoxamine, deferiprone, and deferasirox, in the reactivation of EBV in EBV-positive GC and NPC. We also investigated the cellular consequences following lytic cycle reactivation and the combinatorial effects between the chemicals and ganciclovir.

## 2. Results

### 2.1. Effects of C7 and Its Analog Compounds on EBV Lytic Cycle Reactivation

**Figure 1:**
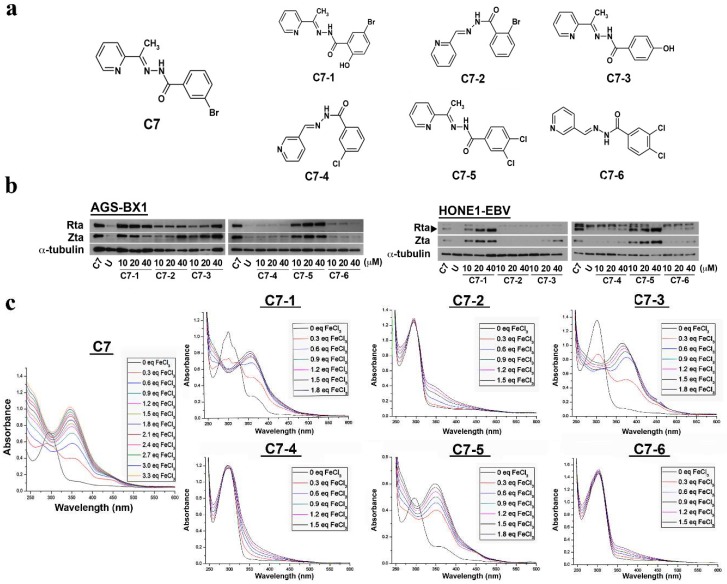
Effects of C7 and its structural analogs on Epstein–Barr virus (EBV) lytic cycle reactivation and the corresponding binding affinity of these compounds to Fe(III). (**a**) The chemical structures of C7 and its analogs obtained from ChemBridge Corp. (**b**) AGS-BX1 and HONE1-EBV cells were either untreated (U) or incubated with the C7 analogs at 10, 20 and 40 μM for 48 h and the expression of IE lytic proteins, Zta and Rta, was detected by Western blotting. Cellular α-tubulin was detected as a loading control. Treatment of AGS-BX1 cells with 10 μM C7 and HONE1-EBV cells with 5 μM C7 were included as positive controls. (**c**) Metal salt FeCl_3_ was added to 50 μM of C7 and C7 analogs from C7-1 to C7-6 in methanol in increments of 0.3 equivalents. Absorbance in the UV-Vis was measured after each addition until saturation was reached. Binding between C7/C7 analogs and FeCl_3_ was indicated by shift in peaks of the absorbance curve.

Available analog compounds of C7 were obtained from ChemBridge Corp. (Figure 1a) and their lytic-inducing ability was assessed in AGS-BX1 and HONE1-EBV cells (GC and NPC cells, respectively, infected with recombinant Akata EBV containing a green fluorescent protein (GFP), open reading frame, and a neomycin resistant gene) by Western blotting. In AGS-BX1 cells, only C7-4 and C7-6 failed to induce any expression of EBV Immediate Early (IE) proteins Zta and Rta at all three concentrations tested (Figure 1b). Further dose optimization experimentation in AGS-BX1 cells revealed that among the four lytic-inducing analogs, C7-2 could only induce weak expression of EBV lytic proteins Zta and Rta whilst C7-1, C7-3 and C7-5 were able to induce strong expression of lytic proteins at concentrations comparable to that of C7 (Figure 1b). Lytic induction by the six analogs was also examined in HONE1-EBV cells at 10, 20, and 40 μM, respectively (Figure 1b), and no expression of EBV lytic proteins was observed after treatment with C7-2, C7-4, and C7-6. Unlike the results observed in AGS-BX1, C7-3 only barely induced the expression of Zta even at high concentration (i.e., 40 μM) whereas C7-1 and C7-5 were more potent in inducing the expression of Zta and Rta in HONE1-EBV cells. Our data showed that among the six C7 analogs, C7-1 and C7-5 were able to potently induce lytic cycle in both GC and NPC cells whereas C7-3 could moderately induce the lytic cycle.

### 2.2. Binding of C7 and Its Lytic-Inducing Analogs to Iron

We showed in the previous section that only some of the C7 analogs could induce EBV lytic reactivation. Since there is structural similarity between C7 and Dp44mT [11], we tested the iron binding capacity of C7 and all the C7 analogs to examine if there is correlation between iron binding capacity and lytic induction ability. The iron binding capacity of these compounds was investigated by UV–Visible (UV–Vis) spectrometry. Binding of iron to these compounds would cause changes in the absorbance in the UV–Vis region, thus a shift of the absorbance is expected in the UV–Vis spectra (Figure 1c). Among all the C7 analogs, C7-2, C7-4, and C7-6, which could not or only weakly induced EBV lytic cycle in either AGS-BX1 or HONE1-EBV cells, had no interaction with iron whilist analogs C7-1, C7-3, and C7-5, which could potently induce lytic cycle, interacted with iron with high affinity (Figure 1c). These findings suggested a correlation between the lytic-inducing ability and the iron binding capacity of C7 as well as its analogs (Table S1).

### 2.3. EBV Lytic Reactivation by Other Clinically Available Iron Chelators

**Figure 2:**
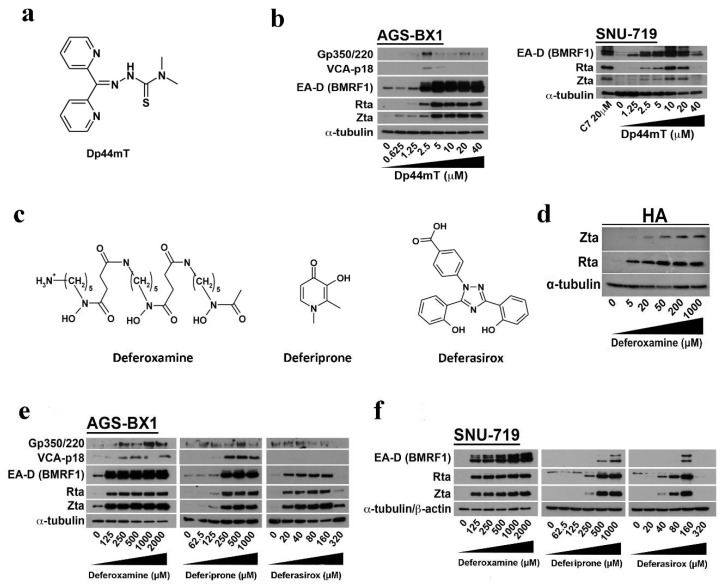
EBV lytic reactivation by clinical iron chelators which are structurally distinct from C7. (**a**) The structure of Di-2-Pyridyl Ketone 4, 4-Dimethyl-3-Thiosemicarbazone (Dp44mT). (**b**) AGS-BX1 and SNU-719 cells were incubated with Dp44mT at 0.625, 1.25, 2.5, 5, 10, 20, and 40 μM for 48 h and the expression of IE lytic proteins, Zta and Rta, early lytic protein EA-D, and late lytic proteins, Gp350/220 and VCA-p18, was detected by Western blotting. Treatment of SNU-719 cells with 20 μM C7 was included as positive control. (**c**) The structures of deferoxamine, deferiprone, and deferasirox, respectively. (**d**) HA cells were incubated with deferoxamine at 5, 20, 50, 200, and 1000 μM, and the expression of Zta and Rta was detected by Western blotting. (**e**,**f**) AGS-BX1 and SNU-719 cells were incubated with deferoxamine at 125, 250, 500, 1000 and 2000 μM; deferiprone at 62.5, 125, 250, 500, and 1000 μM; deferasirox at 20, 40, 80, 160, and 320 μM for 48 h and the expression of IE lytic proteins, Zta and Rta, early lytic protein EA-D, and late lytic proteins, Gp350/220 and VCA-p18, was detected by Western blotting. Cellular α-tubulin was detected as a loading control.

Given that C7 and Dp44mT share some similarities in their structures (Figure 2a), we tested whether Dp44mT could also induce EBV lytic reactivation. We observed increased expression of IE lytic proteins, Zta and Rta, and Early (E) lytic protein, EA-D (BMRF1 gene product), in AGS-BX1 and SNU-719 cells (a GC cell line containing native EBV genome) upon treatment with increasing concentrations of Dp44mT (Figure 2b). Significant expression of these lytic proteins was observed at 5 μM Dp44mT. We wondered if the lytic induction was dependent on the structure or simply caused by intracellular iron chelation by these compounds. To address this question, we further tested whether other structurally distinct clinically available iron chelators including deferoxamine, deferiprone, and deferasirox (Figure 2c) could also induce EBV lytic reactivation. Although a higher dosage of deferoxamine (125–2000 μM), deferiprone (125–1000 μM), and deferasirox (20–320 μM) was needed to induce a comparable expression level of Zta, Rta, and EA-D as that induced by C7 (Figure 2d–f), these compounds could induce the EBV lytic cycle in HA (a recombinant Akata EBV infected NPC cell line), AGS-BX1, and SNU-719 cells, indicating that intracellular iron chelation was likely the trigger in inducing EBV lytic cycle.

### 2.4. Iron Chelation by C7 and Other Iron Chelators Is Required for EBV Lytic Reactivation

**Figure 3:**
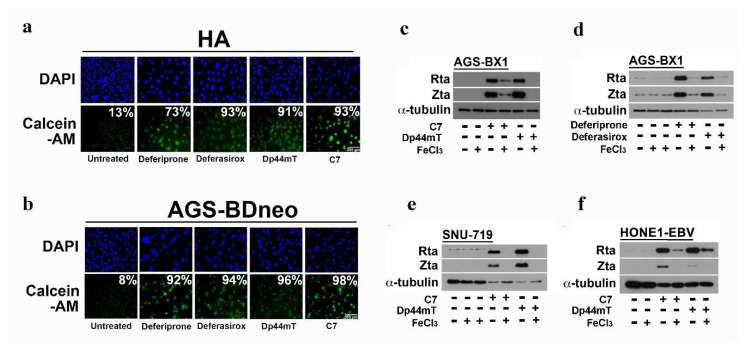
Intracellular iron chelation is required for EBV lytic reactivation by C7 and iron chelators. (**a**,**b**) HA and AGS-BDneo cells were incubated with 1000 μM deferiprone, 1000 μM deferasirox, 40 μM Dp44mT, and 40 μM C7 for 48 h. Culture medium was removed and the cells were incubated with 0.5 μM Calcein-AM, 20 mM HEPES, and 1 mg/mL BSA in PBS for 10 mins at 37 °C. Intensity of green signals, which indicates the level of intracellular iron chelation, was analyzed by immunofluorescence staining. DAPI stained cell nuclei. Blue: Cell nucleus; Green: Calcelin-AM signal. Scale Bar: 250 μm. (**c**,**d**) AGS-BX1 cells were incubated with 10, 80 and 500 μM FeCl_3_; 20 μM C7 and C7-Fe complex; 10 μM Dp44mT and Dp44mT-Fe complex; and 80 μM deferasirox and deferasirox-Fe complex for 48 h and the expression of Zta and Rta was examined by Western blotting. (**e**) SNU-719 cells were incubated with 10 and 20 μM FeCl_3_, 20 μM C7 and C7-Fe complex, and 10 μM Dp44mT and Dp44mT-Fe complex for 48 h and the expression of Zta and Rta was examined by Western blotting. (**f**) HONE1-EBV cells were incubated with 10 μM FeCl_3_, 20 μM C7 and C7-Fe complex, and 10 μM Dp44mT and Dp44mT-Fe complex for 48 h and the expression of Zta and Rta was examined by Western blotting. Cellular α-tubulin was detected as a loading control.

In order to verify that intracellular iron chelation is essential for the induction of EBV lytic cycle, we measured the state of intracellular iron by the well-characterized calcein-AM fluorescent probe. A stronger green fluorescent signal indicates a higher efficiency in chelating intracellular iron by iron chelators [20]. AGS-BDneo (a recombinant Akata EBV-infected GC cell line) and HA cells were treated with either deferiprone, deferasirox, Dp44mT, or C7 for 48 h and then incubated with calcein-AM for 10 mins. Significant (i.e., >70%) green fluorescent signals in C7 or iron chelator-treated cells when compared with those in the untreated controls were observed in both AGS-BDneo and HA cell lines (Figure 3a,b).

Next we tested whether intracellular iron chelation by C7 and other iron chelators is required for the induction of EBV lytic cycle in different cell lines. We precomplexed C7 and other iron chelators with Fe(III) to abrogate their ability to bind intracellular iron and measured the expression of Zta and Rta after treatment with either the original or the precomplexed compounds. A significant reduction in the expression of Zta and Rta in cells treated with the iron complexed C7 and other iron complexes was observed (Figure 3c–f). Supplementation of Fe(III) alone to the cells has no effect on the expression of lytic proteins. These data indicated that the chelation of intracellular iron by C7 and other iron chelators is required for EBV lytic induction.

### 2.5. Enrichment of the Hallmark Gene Set in Hypoxia Signaling Pathway by C7

**Figure 4:**
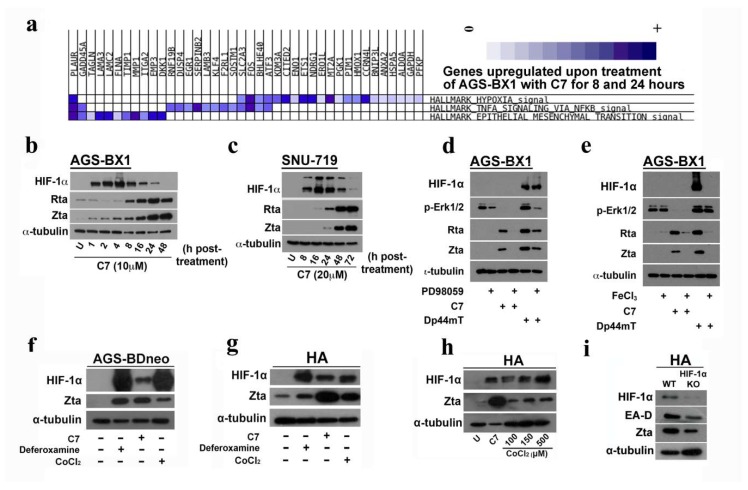
EBV lytic reactivation by C7 requires the activation of ERK and hypoxia signaling pathways. (**a**) Gene sets enriched with a false discovery rate (FDR) smaller than 0.05 were subject to leading edge analysis to obtain a leading edge subset of genes. Clustered map of leading edge subsets upregulated in C7_24 h treatment compared to solvent control. Degree of upregulation is shown with color intensity (darker color indicates greater fold change). (**b**) AGS-BX1 cells were incubated with 10 μM C7 for 1, 2, 4, 8, 16, 24 and 48 h and the expression of Zta, Rta, and HIF-1α was examined by Western blotting. (**c**) SNU-719 cells were incubated with 20 μM C7 for 8, 16, 24, 48 and 72 h and the expression ofZta, Rta, and HIF-1α was examined by Western blotting. (**d**) AGS-BX1 cells were pretreated with 50 μM PD98059 (mitogen-activated protein kinase kinase (MEK) inhibitor) for 1 h, and then incubated with 10 μM M C7 or Dp44mT for 48 h. The expression of Zta, Rta, p-ERK1/2, and HIF-1α was analyzed by Western blotting. (**e**) AGS-BX1 cells were treated with either iron-precomplexed C7 or Dp44mT for 48 h. The expression of Zta, Rta, p-ERK1/2 and HIF-1α was analyzed by Western blotting. (**f**) AGS-BDneo cells were either untreated or treated with 1000 μM deferoxamine, 20 μM C7, or 150 μM CoCl_2_ for 48 h. The expression of Zta and HIF-1α was analyzed by Western blotting. (**g**) HA cells were untreated, or treated with either 1000 μM deferoxamine, 20 μM C7, or 150 μM CoCl_2_ for 48 h. The expression of Zta and HIF-1α was analyzed by Western blotting. (**h**) HA cells were either untreated or treated with either 20 μM C7, 100, 150 or 500 μM CoCl_2_ for 48 h. The expression of Zta and HIF-1α was analyzed by Western blotting. (**i**) Wild-type and HIF-1α knockout HA cells were treated with 20 μM C7 for 24 h. The expression of Zta, EA-D, and HIF-1α was analyzed by Western blotting. Cellular α-tubulin was detected as a loading control.

In order to identify novel pathways involved in the lytic cycle reactivation by C7, we performed a transcriptome profile analysis to analyze the expression of hallmark gene sets in AGS-BX1 cells treated with C7 against vehicle-treated controls. RNA was collected from AGS-BX1 cells treated with DMSO as a solvent control (labeled as U_8 h and U_24 h) and with 10 μM C7 (labeled as C7_8 h and C7_24 h) for either 8 or 24 h, respectively (NCI Geo Datasets accession number: GSE122751). The number of raw reads, reads after quality filtering, reads mapped to human genome (hg19), and reads mapped to EBV genome (NC_007605) are summarized in Table S2. At both 8 h and 24 h timepoints, hallmark gene set in hypoxia signaling was consistently enriched, highlighting the importance of this pathway in C7’s mode of action. Figure 4a shows the leading edge subset genes in clusters for C7_24 h versus U_24 h. Induction of hypoxia-inducible factor-1α (HIF-1α) activity is a key signature of common iron chelators [13] and HIF-1α is a main transcriptional activator controlling a cascade of genes under hypoxic conditions. We measured the expression of HIF-1α and EBV lytic proteins, Zta and Rta, at different time points in AGS-BX1 and SNU-719 cells. The expression of HIF-1α was observed prior to the reactivation of EBV lytic cycle, suggesting the possible involvement of the hypoxia signaling pathway in the lytic cycle reactivation by C7 (Figure 4b,c).

### 2.6. EBV Lytic Reactivation by C7 Requires the Activation of ERK in Addition to Hypoxia Signaling Pathway

Conventional EBV lytic inducers, e.g., TPA and HDAC inhibitors, mediate lytic reactivation through activation of the protein kinase C-delta (PKC-δ) pathway [3,21,22,23,24,25,26,27]. We have previously reported a strong involvement of the ERK1/2 signaling pathway in EBV lytic reactivation by C7 [11]. We therefore examined the possible interaction between the hypoxia and ERK1/2 signaling pathways. Our data showed that there was no observable change in the expression level of HIF-1α in cells treated with ERK1/2 blocker (Figure 4d). However, due to the difference in reaction dynamics of C7 and Dp44mT, the expression of HIF-1α in the C7 treated cells could not be captured at the same time point. These results indicated that there was no chronological relationship between the activation of hypoxia and ERK1/2 signaling pathways. On the other hand, in cells treated with iron-precomplexed Dp44mT, the expression level of p-ERK1/2 decreased, suggesting that iron chelation was required for ERK1/2 activation (Figure 4e). Furthermore, when AGS-BDneo and HA cells were treated with CoCl_2_, which solely stabilized HIF-1α by inactivating Prolyl hydroxylases (PHD) [28], the expression of Zta was significantly lower in these cells than in those treated with C7 and deferoxamine (Figure 4f,g). A similar phenomenon could be observed when HA cells were treated with the maximally available concentration, i.e., 500 μM, of CoCl_2_ (Figure 4h). Although EBV DNA replication was abrogated in HIF-1α knockout (KO) cells upon treatment with C7 (Figure S1), we could still observe an ample amount of Zta and EA-D in these knockout (KO) cells (Figure 4i), suggesting that EBV lytic reactivation could be achieved independent of the HIF-1α pathway. Taken together, the data showed that in addition to the hypoxia signaling pathway, alternative pathway(s) involving ERK activation is required for EBV lytic reactivation by C7 and other iron chelators.

### 2.7. Intracellular Iron Chelation Leads to the Induction of the ERK-Autophagy Axis and Reactivation of EBV Lytic Cycle

**Figure 5:**
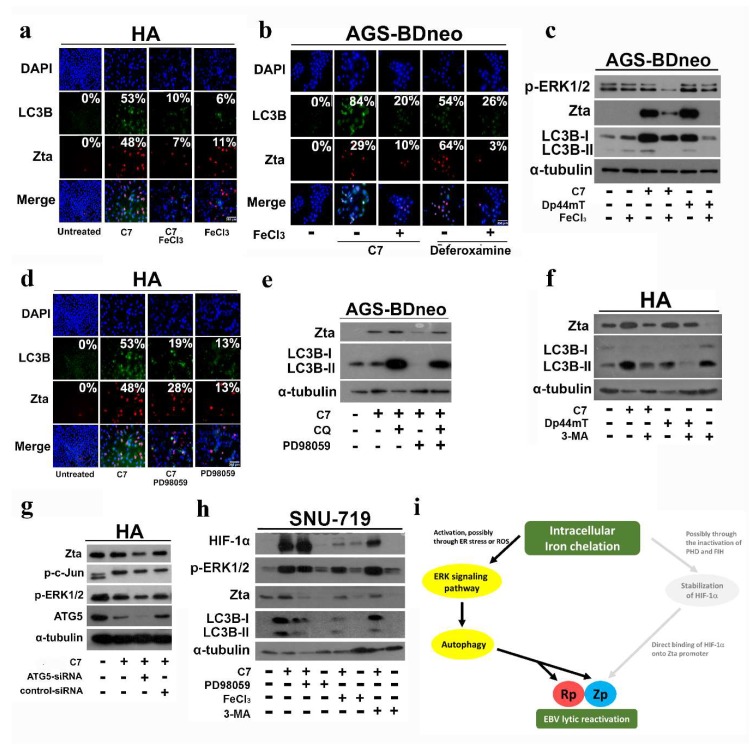
C7 and iron chelators reactivate EBV lytic cycle via intracellular iron chelation and activation of the ERK-autophagy axis. (**a**) HA cells were incubated with either 20 μM C7 or 20 μM iron-precomplexed C7 for 48 h. Expression of Zta (red signals) and LC3B (green signals) was analyzed by immunofluorescence staining. DAPI (blue signals) stained cell nuclei. Scale Bar: 250 μm. (**b**) AGS-BDneo cells were incubated with either 20 μM C7, 20 μM iron-precomplexed C7, 1000 μM deferoxamine, or 1000 μM iron-precomplexed deferoxamine for 48 h. Expression of Zta (red signals) and LC3B (green signals) was analyzed by immunofluorescent staining. DAPI (blue signals) stained cell nuclei. Scale Bar: 250 μm. (**c**) AGS-BDneo cells were treated with either 20 μM C7, 20 μM iron-precomplexed C7, 20 μM Dp44mT, or 20 μM iron-precomplexed Dp44mT for 48 h. The expression of Zta and LC3B was analyzed by Western blotting. (**d**) HA cells were incubated with either 20 μM C7 or 20 μM C7 in combination with 50 μM PD98059 (MEK inhibitor) for 48 h. Expression of Zta (red signals) and LC3B (green signals) was analyzed by immunofluorescent staining. DAPI (blue signals) stained cell nuclei. Scale Bar: 250 μm. (**e**) AGS-BDneo cells were treated with either 20 μM C7, 20 μM C7 in combination with 10 μM chloroquine, 20 μM C7 in combination with 50 μM PD98059 (MEK inhibitor), 20 μM C7 in combination with 50 μM PD98059 (MEK inhibitor), or 10 μM chloroquine for 48 h. The expression of Zta and LC3B was analyzed by Western blotting. (**f**) HA cells were treated with either 20 μM C7, 20 μM C7 in combination with 5mM 3-MA, or 20 μM Dp44mT or 20 μM Dp44mT in combination with 5 mM 3-MA for 48 h. The expression of Zta and LC3B was analyzed by Western blotting. (**g**) Wild-type, *atg5* knockdown and scramble control knockdown HA cells were treated with 20 μM C7 for 48 h. The expression of phosphorylated-c-Jun, phosphorylated-ERK1/2, ATG5, and Zta was analyzed by Western blotting. (**h**) SNU-719 cells were treated with either 20 μM C7, 20 μM C7 in combination with 50 μM PD98059, 50 μM PD98059 alone, 20 μM iron-precomplexed C7, 20 μM C7 in combination with 3-MA, or 3-MA alone for 48 h. The expression of HIF-1α, p-ERK1/2, Zta, and LC3B was analyzed by Western blotting. (**i**) Schematic illustration of EBV lytic reactivation via the proposed intracellular iron chelation-ERK-autophagy axis.

As it has been reported that iron chelation and ERK1/2 activation can both regulate the autophagy machinery [15,16,29,30], we wonder if autophagy is involved in this pathway. HA cells were treated with either C7, deferoxamine, Dp44mT, or their iron-precomplexed counterparts. A significantly lower expression of Zta was observed in cells treated with the iron-precomplexed C7 when compared to cells treated with C7 (48% vs. 7%). Similar effect was also observed in LC3B puncta formation (53% vs. 10%) (Figure 5a). Results obtained from AGS-BDneo cells showed a similar observation, in which cells treated with the iron-precomplexed C7 showed significantly lower expression of Zta (29% vs. 10%) and LC3B puncta (84% vs. 20%). Cells treated with iron-precomplexed deferoxamine also showed significantly lower expression of Zta (64% vs. 3%) and LC3B puncta (54% vs. 26%) (Figure 5b). It was further verified in Western blot analysis that the expression levels of Zta, LC3B and ERK1/2 were significantly lower in AGS-BDneo cells treated with iron-precomplexed Dp44mT and C7 (Figure 5c). The above data support that intracellular iron chelation is required for ERK1/2 activation and autophagy initiation. We also observed that phosphorylated ataxia telangiectasia-mutated (ATM) level was decreased in cells treated with iron-precomplexed C7 or deferoxamine (Figure S2).

ERK1/2 activation was found to promote autophagy in hepatocellular carcinoma and colon cancer cells [29,30]. In order to understand their relationship in the EBV-positive epithelial cells, HA cells were treated with a combination of an ERK inhibitor and C7, and tested for the induction of autophagy and lytic reactivation. We could observe a significant decrease in Zta expression level (48% vs. 28%) and LC3 puncta formation (53% vs. 19%) (Figure 5d). The same phenomenon was also observed in AGS-BDneo cells by Western blot analysis (Figure 5e), suggesting that ERK1/2 activation is required for EBV lytic reactivation possibly via initiation of autophagy. We further tested if autophagy initiation is required for EBV lytic reactivation in cells treated with C7 or iron chelators. HA cells treated with a combination of 3-MA and either C7 or Dp44mT showed a significantly lower expression level of Zta than those treated with C7 or Dp44mT alone (Figure 5f). A similar phenomenon was observed in *atg5* knockdown cells (Figure 5g). Furthermore, inhibition of autophagy could abrogate viral DNA replication induced by C7 (Figure S3), supporting the hypothesis that autophagy initiation by C7 or iron chelators may be linked to EBV lytic reactivation. We set out to verify the mechanisms of EBV lytic reactivation through intracellular iron chelation in SNU 719 cells which harbor native EBV genomes. First, ERK signaling pathway has no relationship with HIF-1α pathway as HIF-1α protein levels remained unchanged in ERK-inhibited cells upon C7 treatment (refer to Figure 5h, lanes 2 and 3). Second, expression of ERK1/2, Zta, and LC3B was abrogated in cells treated with iron-precomplexed C7, inferring that intracellular iron chelation is upstream of ERK signaling, autophagy, and EBV lytic reactivation (refer to Figure 5h, lanes 2 and 5). Third, expression of Zta was abrogated while the ERK1/2 level remained unchanged in autophagy-inhibited cells, suggesting that autophagy acts upstream of EBV lytic reactivation and downstream of the ERK1/2 signaling pathway (refer to Figure 5h, lanes 2,7). Furthermore, in an attempt to identify the specific lytic promoter activated upon C7 treatment, a luciferase expressing plasmid under the control of either a Z or R promoter was transfected into HA and AGS-BDneo cells, which were then treated with C7 for 4, 8, and 12 h (Figure S4). In both HA and AGS-BDneo cells, we could observe a gradual increase in the activities of Z promoter with increasing duration of treatment while the activites of R promoter were detectable but insignificant. These results indicated that the activation of Z promoter is dominant over R promoter upon C7 treatment. Interestingly, C7 could not induce a complete viral lytic reactivation as late lytic proteins such as gp350 and VCA-p18 could not be detected by either Western blot or immunofluorescent staining (Figure S5). Taken together, a novel mechanism of EBV lytic reactivation via intracellular iron chelation-ERK-autophagy axis is demonstrated(Figure 5i).

### 2.8. C7 Sensitizes EBV-Positive Gastric Carcinoma Cells to Killing by Ganciclovir

**Figure 6:**
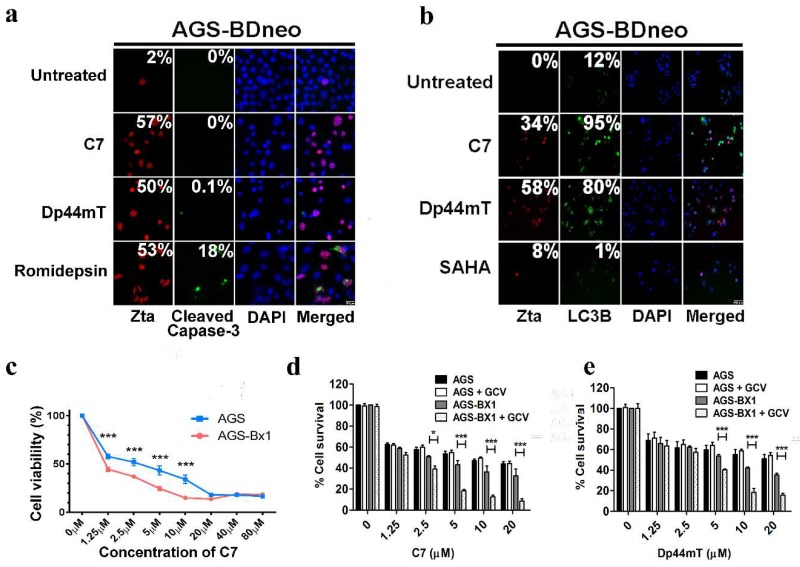
C7 induces cell death via a caspase-independent autophagy-dependent mechanism and both C7 and Dp44mT confer susceptibility of AGS-BX1 cells to ganciclovir. (**a**) AGS-BDneo cells were incubated with either 20 μM C7, 20 μM Dp44mT, or 5 nM romidepsin for 48 h. Expression of Zta (red signals) and cleaved caspase-3 (green signals) was analyzed by immunofluorescent staining. DAPI (blue signals) stained cell nuclei. Scale Bar: 100 μm. (**b**) AGS-BDneo cells were incubated with either 20 μM C7, 20 μM Dp44mT, or 10 μM SAHA for 48 h. Expression of LC3B (green signals) and Zta (red signals) was analyzed by immunofluorescent staining. DAPI (blue signals) stained cell nuclei. Scale Bar: 250 μm. (**c**) AGS and AGS-BX1 cells seeded in triplicate in 96-well plates were treated with 0, 1.25, 2.5, 5, 10, 20, 40, or 80 μM C7 for 48 h. An 3-(4,5-Dimethylthiazol-2-yl)-2,5-diphenyltetrazolium bromide (MTT) solution was added into each well and wells were incubated in a 37 °C, 5% CO_2_ incubator for 5 h. OD430 nm and OD570 nm were measured and cell viability was plotted. (**d**,**e**) EBV-negative and -positive GC (AGS and AGS-BX1) cells were treated with either increasing concentrations of C7 or Dp44mT, 10 μg/mL GCV or their combination for 7 days. The number of viable GC cells upon C7 and Dp44mT treatment was determined by trypan blue exclusion assay. Results are presented as percentages of viable cell populations among treated cells compared with those of untreated control. Error bars represent the standard error of mean (SEM) of data obtained in at least three independent experiments. * *p* ≤ 0.05; ***: *p* ≤ 0.001.

HDAC inhibitors, including romidepsin and suberoylanilide hydroxamic acid (SAHA), were shown to induce caspase-dependent cell death after EBV lytic reactivation [2,5]. We wonder if C7 and other clinically available iron chelators can also induce caspase-dependent cell death in EBV-positive epithelial cancers. AGS-BDneo cells were treated with either C7, Dp44mT, or romidepsin for 48 h and examined for the percentage of cells expressing cleaved caspase-3 by immunofluorescent staining. However, we could not observe any signal of cleaved caspase-3 in the AGS-BDneo cells upon C7 treatment, contrary to the expression of cleaved caspase-3 in the cells treated with romidepsin (i.e., 0% vs. 18%) (Figure 6a and Figure S6). We postulated that autophagy rather than apoptosis might be involved in the cell death mechanism of C7. Indeed, LC3B punta formation was observed in a higher percentage of AGS-BDneo cells upon treatment with either C7 or Dp44mT than that with SAHA (i.e., 95% vs. 80% vs. 1%) (Figure 6b). In contrast, cleaved caspase-3 and cleaved caspase-7 could only be detected in cells treated with romidepsin but not with C7 and Dp44mT (Figure S6).

Finally, we tested whether lytic cycle reactivation by C7 or Dp44mT could confer susceptibility of EBV-positive epithelial cancers to killing by ganciclovir (GCV). AGS-BX1 cells and their EBV-negative counterparts, AGS cells, were pretreated with GCV followed by treatment with increasing concentrations of C7 or Dp44mT for 48 h. The percentages of proliferating cells in these conditions were determined by trypan blue exclusion assay. Treatment with either C7 or Dp44mT alone was sufficient to achieve enhanced killing of EBV-positive cells in a dose-dependent manner when compared to that of their EBV-negative counterparts (Figure 6c). Interestingly, when the cells were treated with a combination of either C7 or Dp44mT with GCV, significantly enhanced killing of EBV-positive cells could be observed (*t*-test; *p* < 0.001). Despite the induction of autophagy (instead of apoptosis), reactivation of EBV lytic cycle by C7 could confer susceptibility of EBV-positive epithelial cancers to the cytotoxic effect of GCV (Figure 6d,e).

## 3. Discussion

Lytic induction therapy offers specific killing of Epstein–Barr virus (EBV)-positive malignancies and has been tested in clinical trials [10]. However, the efficacy of lytic induction by clinically available EBV lytic inducers, such as histone deacetylase (HDAC) inhibitors, is limited by the refractoriness of cells to EBV lytic reactivation [4,9].

We identified new classes of lytic inducers through a high throughput screening of more than 50,000 chemical compounds in which C7 was selected for further study due to its ability to induce the EBV lytic cycle in a large panel of EBV-positive epithelial cancer cell lines [11]. Interestingly, C7 contains a transition metal binding moiety (Figure 1a) and has structural similarity to Dp44mT, a known chelator of intracellular iron (Figure 2a). The testing of C7 analogs revealed that only those that interacted with iron could reactivate EBV lytic cycle in EBV-positive epithelial cancer cells. We subsequently showed that Dp44mT and the other clinically available iron chelators such as deferoxamine, deferiprone, and deferasirox could also reactivate EBV lytic cycle in EBV-positive epithelial cancer cells (Figure 2). We further demonstrated that intracellular iron chelation was required for the iron-chelating compounds to reactivate EBV lytic cycle, as precomplexing these compounds to Fe(III) ions prior to cell treatment greatly diminished their ability to reactivate EBV lytic cycle (Figure 3). Taken together, intracellular iron chelation in EBV-positive epithelial cells might be the major upstream signaling component which leads to the reactivation of EBV lytic cycle.

To gain further insight into the novel mechanism of EBV lytic cycle reactivation by C7, we performed transcriptome profile analysis which revealed enrichment in the expression of genes in the hypoxia signaling pathway. HIF-1α was further confirmed to be stabilized upon C7 treatment (Figure 4). One possible explanation for the stabilization of HIF-1α upon intracellular iron chelation by C7 treatment could be related to the inactivation of two iron-interacting proteins, prolyl hydroxylases (PHD) and factors inhibiting HIF-1α (FIH), which are involved in the degradation and inactivation of HIF-1α, respectively [31,32]. The direct linkage between HIF-1α and EBV lytic reactivation was shown in a recent report demonstrating that HIF-1α could directly bind the Z promoter [33]. However, our data showed that the sole stabilization of HIF-1α does not induce a comparable expression level of Zta in cells treated with C7 or other iron chelators. Furthermore, an ample amount of Zta can be detected in HIF-1α knockout cells (Figure 4), suggesting that other pathways might be involved in EBV lytic reactivation by C7 and other iron chelators.

We have shown previously that only pharmacological inhibition of the ERK1/2 pathway, rather than the PI3K, p38 MAPK, PKC-δ, or ATM signaling pathways, could inhibit EBV lytic cycle reactivation by C7 [11]. In this study, we examined potential chronological relationship between the hypoxia and ERK1/2 signaling pathways and found that the two pathways operate independently of each other (Figure 4), suggesting alternative pathway(s), which require ERK activation, may be involved in EBV lytic reactivation by C7 and other iron chelators. Iron chelation has been shown to activate ERK1/2 signaling pathway. For instance, deferoxamine has been shown to activate p38 and ERK1/2 in colon epithelial cells and oral keratinocytes [34,35]. Consistently, our data showed that precomplexing with iron could reduce phosphorylation of ERK1/2 and induction of EBV lytic cycle by C7 or other iron chelators (refer to Figure 5), suggesting that C7 reactivates EBV lytic cycle through the chelation of intracellular iron with subsequent ERK1/2 activation.

Iron chelation and ERK1/2 activation have been reported to initiate autophagy [15,16,29,30]. Interestingly, many recent studies have reported that viruses can manipulate autophagy machinery for different viral processes [17,18,19]. In the context of EBV, it was found that during EBV replication in several cell types, the virus hijacks autophagic vesicles to promote its intracellular transportation and viral production via the blockage of autophagy at the final degradative step [36]. In B cells, immediate early lytic protein, Rta, initiates the transcription of genes that participate in the formation of autophagosomes and regulation of autophagy. Autophagy is also thought to be critical to EBV lytic reactivation and production of viral particles in the same study [37]. Our data showed that autophagy was required for EBV lytic reactivation since expression of Zta was abrogated in autophagy-inhibited cells. In addition, autophagy initiation was mediated by iron chelation and ERK activation as autophagy was inhibited in cells treated with C7 precomplexed with iron and ERK1/2 blockers, respectively (refer to Figure 5), indicating the chronological sequence of iron chelation, ERK activation, autophagy initiation, to EBV lytic reactivation in EBV-positive epithelial cells. Moreover, the measurement of viral DNA replication in autophagy-inhibited cells supports an essential role of autophagy in viral DNA replication (Figure S3).

Despite the potent ability in inducing the expression of early lytic proteins as well as viral DNA replication, C7 could not induce a complete viral lytic reactivation as late lytic proteins were not detected with increasing dosage of C7 over a prolonged period of treatment (Figure S1). On the contrary, HDAC inhibitors, including SAHA and romidepsin, could induce strong expression of EBV late lytic proteins and production of infectious viral particles in the EBV-positive epithelial cells [2,4,5]. Metal ion, e.g., zinc, is indispensable for some viral proteins and plays essential role in the survival and pathogenesis of different viruses [38]. In the context of virion production, zinc ion has been shown to interact with the nucleocapsid protein of HIV-1 [39] and a possible zinc binding domain in the tegument protein UL94 of CMV [40], and is essential for the formation of a functional envelope glycoprotein complex of Junín Virus [41]. Since C7 and other iron chelator could potentially interact with zinc, chelation of intracellular zinc ion might explain the failure to produce late lytic proteins upon C7 treatment. 

In summary, we have discovered a novel mechanism of reactivation of EBV lytic cycle through intracellular iron chelation and induction of ERK-autophagy axis in EBV-positive epithelial malignancies, raising the question whether clinically available iron chelators can be incorporated into existing therapeutic regimens to treat these cancers.

## 4. Materials and Methods

### 4.1. Cell Lines and Culturing Conditions

AGS, AGS-BDneo, and AGS-BX1 were gifts from Prof. L. Hutt-Fletcher, Louisiana State University, Baton Rouge, LA, USA. AGS is a Epstein–Barr virus (EBV)-negative gastric adenocarcinoma (GC) cell line; AGS-BDneo and AGS-BX1 are recombinant EBV-infected GC cell lines [42]. SNU-719 is a GC cell line containing native EBV genomes (Korean Cell Line Bank, Seoul, Korea). HONE1-EBV and HA cells (gifts from Prof. George S.W. Tsao of the University of Hong Kong, Hong Kong SAR, China) are recombinant EBV infected Nasopharyngeal carcinoma (NPC) cell lines. The recombinant Akata EBV genomes of AGS-BX1 and HONE1-EBV cells contain both a green fluorescent protein (GFP) open reading frame and a neomycin resistant gene. AGS, AGS-BDneo, and AGS-BX1 cells were maintained in Ham’s F-12 media with 10% FBS, and all other cells in RPMI 1640 media with 10% FBS at 37 °C, 5% CO_2_. All EBV-positive cell lines are maintained in media with 500 μg/mL G418. These cell lines were being authenticated by the 16 Genetic Sites PowerPlex^®^ 16HS services provided by Genetica DNA Laboratories (1440 York Court Burlington, NC 27215, USA). Sixteen short tandem repeat (STR) loci, including D3S1358, D7S820, vWA, FGA, D8S1179, D21S11, D18S51, D5S818, D13S317, D16S539, TH01, TPOX, CSF1PO, AMEL, Penta D, and Penta E, were detected. The STR DNA profile and the electropherogram of the submitted cell lines were provided Genetica (STR profiles of our GC and NPC cell lines are summarized in the attached file). The STR profile of AGS cells is identical to the standard AGS STR profile stated in ATCC (ATCC^®^ CRL-1739™, Manassas, VA, USA). The AGS-BDneo and AGS-BX1 cells are identical to their parental EBV-negative AGS cells with only an additional allele detected at the FGA locus. This minor allelic variation may be due to genetic instability after long-term cell culture. The SNU-719 cell is identical to the standard SNU-719 STR profile stated in the Korean Cell Line Bank. The HONE-1 cell type is identical to the standard HONE1 STR profile stated in the Cellosaurus database except additional alleles detected at D13S317, D7S820, D8S1179, and TH01. The STR profile of HA and HONE1-EBV cells are identical to their parental EBV-negative HONE-1 cells, with only an additional allele detected at D3S1358 locus and a different number of copies detected in one of the allele at D21S11 locus for HONE1-EBV. This minor allelic variation may be due to genetic instability after long-term cell culture. No identical profile was observed when compared the profiles of our cell lines against the known STR profiles of other cell lines available in DSMZ database.

### 4.2. Drug Treatment

Drugs for precomplex experiment were first mixed with FeCl3 in 1:1 molar ratio and incubated at room temperature for 5 mins. Cells were treated at 70% confluence with the drugs or precomplexed drugs at their specified concentrations for 48 h in a 5% CO_2_, 37 °C incubator. Chemical compounds used: deferoxamine (Novartis NDC 0078-0467-91, Basel, Switzerland), deferiprone (ApoPharma, Toronto, ON, Canada), deferasirox (Novartis), Dp44mT (gift from Prof. Des Richardson, University of Sydney, Australia), C7, C7-1, C7-2, C7-3, C7-4, C7-5, C7-6 (ChemBridge, ID#5632947, ID#5636413, ID#6120380, ID#5631431, ID#5335854, ID#5630707, and ID#5636784, respectively), PD98059 (Merck 513000, Kenilworth, NJ, USA), romidepsin (Selleck S3020, Houston, TX, USA), 3-MA (Sigma Aldrich M9281, St. Louis, MO, USA), and chloroquine (Sigma Aldrich C6628).

### 4.3. Calcein-AM Measurement

HA and AGS-BDneo cells grew on coverslips were treated at different dosage for 48 h. Culture medium was removed, and the cells were washed with PBS once. 0.5 μM Calcein-AM (Life Technologies Limited, C1430, Waltham, MA, USA) were mixed with 1 mg/mL BSA and 20 mM HEPES in a total volume of 300 μL. The mixture was added to the treated cells and was incubated for 10 mins in a 37 °C, 5% CO_2_ incubator.

### 4.4. Western Blot Analysis

Protein from the treated cell cultures was extracted and Western blot analysis was performed as described previously [43]. Expression of EBV lytic proteins was detected with anti-Zta (1:200; gift from Prof. P. Farrell, Imperial College, London, UK), anti-Rta (1:1000; Argene, Varilhes, France), anti-BMRF1 (1:1000; gift from Dr.KH Chan, Department of Microbiology, HKU, Hong Kong SAR, China), and anti-VCA p18 (1:500) and anti-gp350/220 anti-bodies (1:500); anti-VCA p18 and anti-gp350/220 antibodies are both gifts from Prof. J. Middeldorp, VU University, Netherlands. Expression of phosphorylated ERK1/2 was detected with p-ERK1/2 rabbit polyclonal antibodies, (1:1000; Cell Signaling Technology, Danvers, MA, USA). Expression of human cellular α-tubulin and β-actin was detected with α-tubulin and β-actin antibody (1:5000; Sigma-Aldrich), respectively, as loading controls.

### 4.5. RNA-Sequencing Analysis

RNA was extracted from treated cells using RNeasy^®^ Plus Mini Kit (Qiagen, Venlo, Netherlands) according to manufacturer instruction. AGS-BX1 cells were seeded in 5 cm plates overnight at 37 °C, 5% CO_2_ to 70% confluence. The cells were treated with DMSO as solvent control or 10 μM C7 for 8 h or 24 h.

### 4.6. Bioinformatics Analysis

Sequence alignment, expression quantification, and differential expression analysis were performed by CGS, HKU. Reads from sequencing were assigned to samples using CASAVA and adapter sequences, reads with more than 5% unknown bases and reads with more than 50% bases of quality score lower than or equal 10 were filtered. Analyses were performed with CLC Genomics Workbench Version 7.0.4 (CLC bio, Aarhus, Denmark). Reads after filtering were aligned to the human genome (hg19) first with default parameters except for similarity fraction (set to 0.9), length fraction (set to 0.8), and strand specific (set to reverse). The unmapped read were then aligned to the EBV genome (NC_007605). Expression quantification was performed separately for human and EBV genes and differential expression analysis was performed with Kal’s test with Bonferroni correction. The differentially expressed genes were subsequently subject to Gene Set Enrichment Analysis (GSEA) with their javaGSEA desktop application (Subramanian et al. 2005). The genes were enriched against the hallmark gene sets in their Molecular Signature Database (MSigDB, v5.0, Broad Institute). Gene sets enriched with a false discovery rate (FDR) < 0.05 were subject to subsequent leading edge analysis to obtain a leading edge subset of genes (NCI Geo Datasets accession number: GSE122751).

### 4.7. Metal Binding Titration, Measurement, and Analysis

Metal ion stock solutions (1.5 mM) were prepared by dissolving the respective metal chloride in distilled water. Hit compound C7 and its analogs respective stock solutions (5 mM) were prepared by dissolving in N,N dimethylformamide (DMF). Methanol was used as a solvent for the measurement. Methanol (3 mL) was first added to a 4 mL cuvette, to which ligand solution (30 μL, 5 mM) was added to constitute a solution of 50 μM concentration. Absorbance of the ligand was measured by UV-Vis spectrometer (Cary^®^ 50 UV-Vis Spectrometer, Agilent Technologies, Santa Clara, CA, USA). Metal ion stock solution was then added to the cuvette in 0.367 equivalents (30 μL, 1.5 mM) with thorough mixing, followed by immediate measurement of absorbance, until saturation was observed. All the absorbance curves obtained from titrating the ligand against a specific metal ion were plotted in a graph to analyse the change in absorbance pattern. 

### 4.8. Immunofluorescence Staining

AGS-BDneo and HA cells grew on cover slips were treated with drugs for specific duration depending on experimental needs. Cells were fixed with acetone for 10 mins at room temperature. The fixed cells were then stained with anti-Zta, cleaved caspase-3, or LC3B rabbit polyclonal antibody (1:200; Cell Signaling Technology, Beverly, MA) overnight at 4 °C. Expression of the proteins was visualized with Alexa Fluor 488 F(ab′)2 fragment of goat anti-rabbit IgG antibody (1:500; Invitrogen) under fluorescence microscopy or Carl Zeiss LSM 710 confocal microscope (Carl Zeiss, Oberkochen, Germany). Nuclei of cells were stained with 4′,6-diamidino-2-phenylindole (DAPI) (Roche, Mannheim, Germany).

### 4.9. MTT Assay

AGS-BX1 and AGS cells (2 × 104 cells/well) were treated with or without ganciclovir (μM) and various concentrations of either C7 or Dp44mT for 48 h. MTT assays were performed as previously described [44].

### 4.10. CRISPR Gene Knockout

gRNA targeting HIF-1α (forward 5′-CACCGGTTATGGTTCTCACAGATGA; reverse 5′-AAACTCATCTGTGAGAACCATAACC) was cloned into pSpCas9(BB)-2A-Puro (PX459) V2.0 (Addgene, #62988, Watertown, MA, USA) according to the supplier’s protocol. Sequence verified plasmid was transfected with Genejuice (Merckmillipore, #70967, Burlington, MA, USA) according to the manufacturer’s protocol. Transfected cells were plated in 96-well plates and were screened in DMEM medium supplemented with 1 µg/mL puromycin (Sigma Aldrich, #P9620-) for 2 weeks to allow clonal expansion. Expanded candidates were trasnfered to 24-well plates where they were verified by HIF-1α detection by Western blot analysis under treatment with C7 and iron chelators.

### 4.11. Transient Gene Knockdown

siRNA targeting *atg5* (Dharmacon, #M-004374-04-0005) and scamble siRNA (Dharmacon, # D-001210-01-05, Lafayette, CO, USA) were transfected with Lipofectamine 2000 (Thermofisher, #11668027-) according to the manufacturer’s protocol. Cells were incubated with transfection mix for 24 h, then the medium was removed. Cells were immediately treated with C7 for another 24 h.

### 4.12. Quantitative PCR Assay

HA and AGS-BDneo cells were treated with different compounds for a destinated duration according to their experimental purposes. After treatment, the cells were pelleted and washed once with PBS. DNA from the cell pellets was extracted according to the manufactuerer’s instructions (DNeasy^®^ Blood & Tissue Kit, Qiagen), and quantitative PCR was performed as described previously [4]. EBV viral load was presented as number of viral genomes per cell normalized to the untreated samples. Data were determined in triplication in a 96-well plate format.

### 4.13. Luciferase Reporter Assay

Luciferase reporter vector (pGL2) under the regulation of Rp and Zp were gifts from Prof Ching-Hwa Tsai, National Taiwan Univerisity, Taiwan. Either of the plasmids were cotransfected with the pTK-renillase internal control in HA and AGS-BDneo cells. After 24 h of incubation, cells were treated either untreated or treated with 20 μM C7 for 24 h. Cell lysates were prepared and assayed for luciferase activity according to the manufacturerer’s instructions (Dual-Glo^®^ Luciferase Assay System, Promega, Madison, WI, USA). Data are presented as relative luciferase activity normalized to the untreated samples

## 5. Conclusions

We discovered a novel mechanism of reactivation of the EBV lytic cycle through intracellular iron chelation and induction of the ERK-autophagy axis in EBV-positive epithelial malignancies, providing scientific rationale to test whether clinically available iron chelators can be incorporated into existing therapeutic regimens to treat these cancers.

## Supplementary Materials

The following are available online at http://www.mdpi.com/2072-6694/10/12/505/s1, Figure S1: C7 could not induce a complete EBV lytic reactivation in wildtype and HIF-1α knock out HA cells, Figure S2: C7 and deferoxamine led to genome instability and DNA breaks in HONE1-EBV cells, Figure S3: Lytic reactivation and DNA replication were halted in AGS-BDneo cells upon treatment with inhibitor of autophagy, Figure S4: Dominant activation of Z promoter over R promoter in HA and AGS-BDneo cells upon treatment with C7, Figure S5: C7 could not induce a complete EBV lytic reactivation despite a strong induction of Zta expression, Figure S6: Apoptosis-independent cell death was involved in HA cells treated with C7, Table S1: Relative strength of EBV lytic induction and Fe(III) binding amongst C7 and its analogs, Table S2: Summary of RNA sequencing data of AGS-BX1 cell line with or without treatment by C7.
