# Supplementary material for: Intracellular Iron Chelation by a Novel Compound, C7, Reactivates Epstein–Barr Virus (EBV) Lytic Cycle via the ERK-Autophagy Axis in EBV-Positive Epithelial Cancers
Source: Cancers (Basel). 2018 Dec 11;10(12):505. doi: 10.3390/cancers10120505 (PMC6316324; doi:10.3390/cancers10120505)
Supplement: Supplementary file 1 [file cancers-10-00505-s001.pdf]

Supplementary materials: Intracellular Iron Chelation by a Novel Compound, C7, Reactivates Epstein–Barr Virus (EBV) Lytic Cycle via the ERK-Autophagy Axis in EBV-Positive Epithelial Cancers

Stephanie Pei Tung Yiu, Kwai Fung Hui, Chung King Choi, Richard Yi Tsun Kao, Chi Wang Ma, Dan Yang and Alan Kwok Shing Chiang

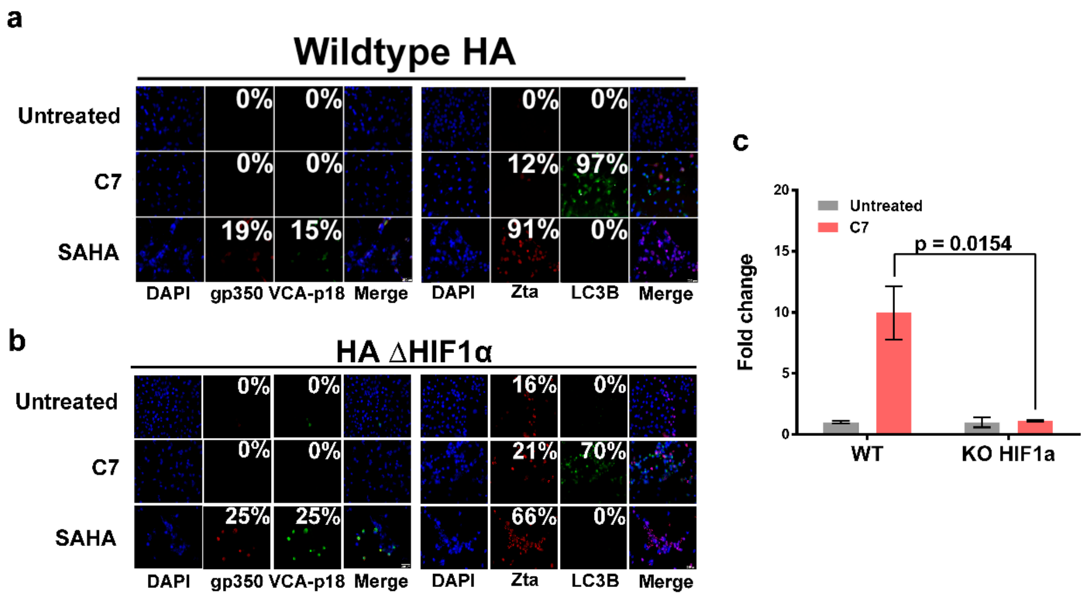

Figure S1. C7 could not induce a complete EBV lytic reactivation in wildtype and HIF-1α knock out HA cells. (**a**) HA cells and (**b**) HIF-1α knock out HA cells were incubated with either 20 μM C7 or 10 μM SAHA for 72 h. Expressions of Zta, LC3B, VCA-p18 and gp350 were analyzed by immunofluorescence staining. DAPI (blue signals) stained cell nuclei. Scale Bar: 250 μm. (**c**) Quantitative PCR showing the induction of EBV DNA replication in wild type and HIF-1α knock out HA cells either untreated or treated with 20 μM C7 for 24 h. Data are presented as number of viral genomes per cell normalized to the untreated sample. Error bars show standard errors of triplicate wells.

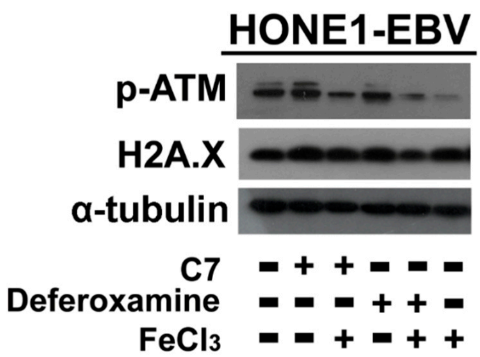

Figure S2. C7 and deferoxamine led to genome instability and DNA breaks in HONE1-EBV cells. HONE1-EBV cells were either untreated or treated with 20 μM C7, 20 μM iron-precomplexed C7, 1000 μM deferoxamine, 1000 μM iron-precomplexed deferoxamine or iron alone for 48 h. Expressions of phosphorylated ATM and H2A.X were analyzed via Western blot.

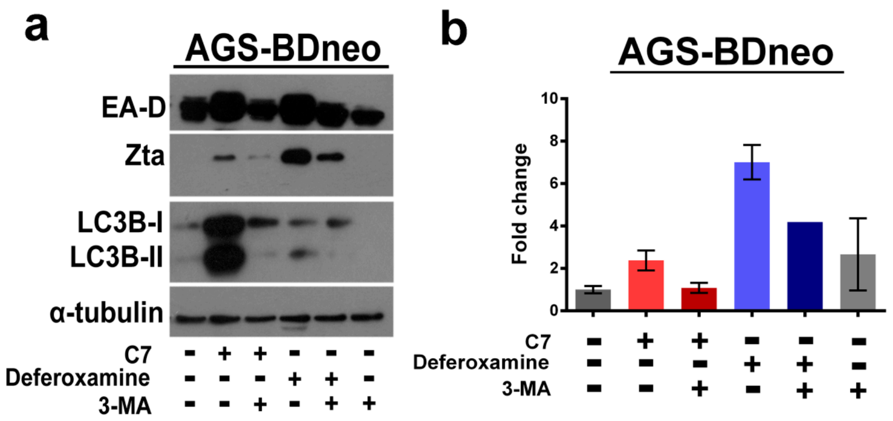

Figure S3. Lytic reactivation and DNA replication were halted in AGS-BDneo cells upon treatment with inhibitor of autophagy. (**a**) AGS-BDneo cells were either untreated or treated with either 20 μM C7, 20 μM C7 in combination with 5 mM 3-MA, 1000 μM deferoxamine, 1000 μM deferoxamine in combination with 5mM 3-MA or 3-MA alone for 48 h. Expressions of EA-D, Zta and LC3B were analyzed via Western blot. (**b**) Quantitative PCR showing the induction of EBV DNA replication in untreated or AGS-BDneo cells treated with either 20 μM C7, 20 μM C7 in combination with 5mM 3-MA, 1000 μM deferoxamine, 1000 μM deferoxamine in combination with 5mM 3-MA or 3-MA alone for 48 h. Data are presented as number of viral genomes per cell normalized to the untreated sample. Error bars show standard errors of triplicate wells.

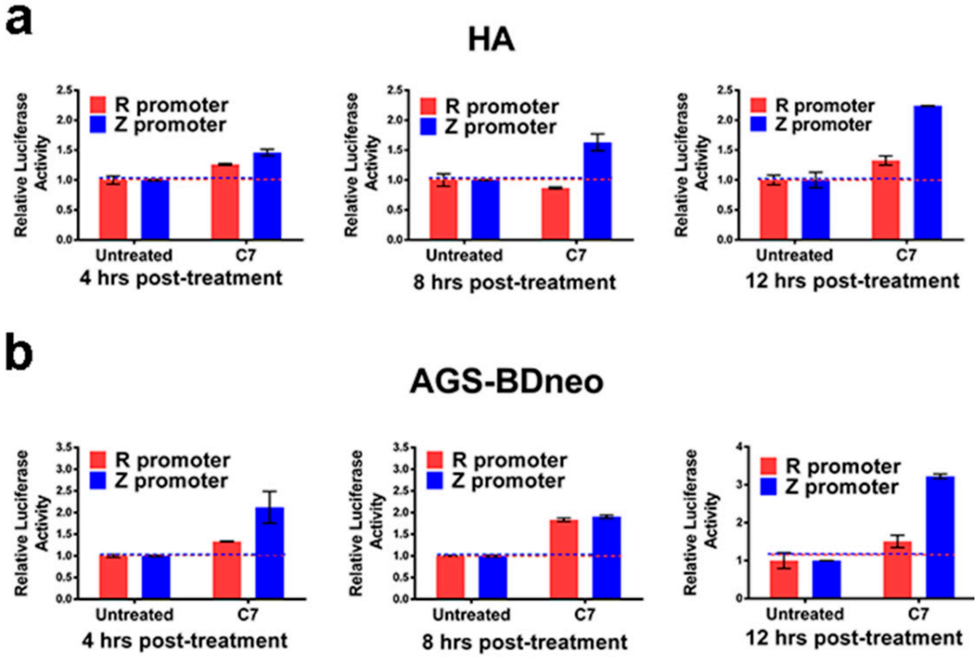

Figure S4. Dominant activation of Z promoter over R promoter in HA and AGS-BDneo cells upon treatment with C7. (**a**) HA and (**b**) AGS-BDneo cells were cotransfected with either of the luciferase reporter vector (pGL2) under the regulation of Rp and Zp with the pTK-renillase internal control for 24 h. The transfected cells were then either untreated or treated with 20 μM C7 for 4, 8 and 12 h. Cell lysates were prepared and assayed for luciferase activity. Data are presented as relative luciferase activity normalized to the untreated samples. Error bars show standard errors of triplicate wells.

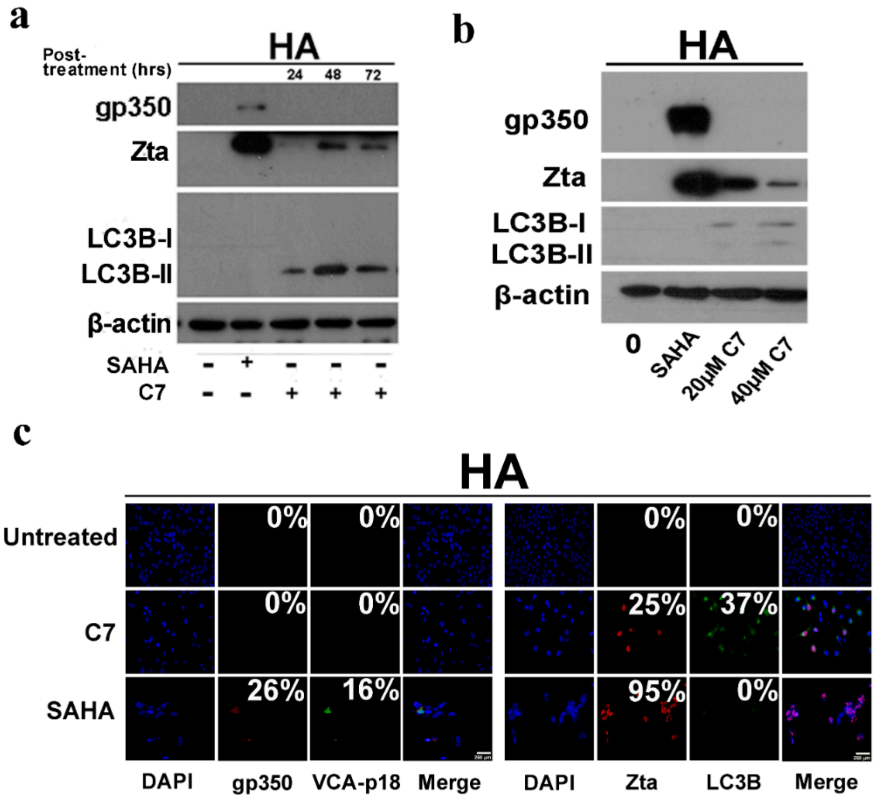

Figure S5. C7 could not induce a complete EBV lytic reactivation despite a strong induction of Zta expression. (**a**) HA cells were either untreated or treated with either 20 µM C7 or 10 µM SAHA for 24, 48 or 72 h. Expressions of gp350, Zta and LC3B were analyzed via Western blot. (**b**) HA cells were either untreated or treated with either 20 µM C7, 40 µM C7 or 10 µM SAHA for 482 h. Expressions of gp350, Zta and LC3B were analyzed via Western blot. (**c**) HA cells were either untreated or incubated with either 20 µM C7 or 10 µM SAHA for 72 h. Expressions of Zta, LC3B, VCA-p18 and gp350 were analyzed by immunofluorescence staining. DAPI (blue signals) stained cell nuclei. Scale Bar: 250 µm.

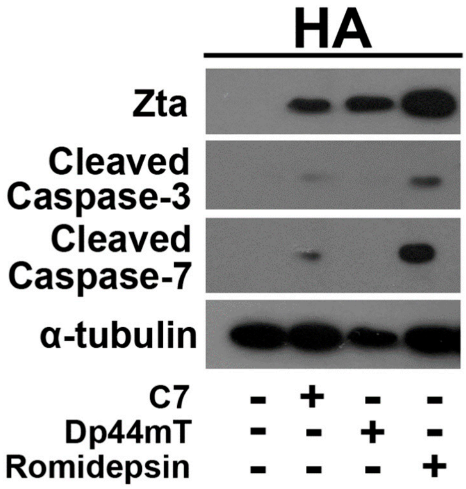

Figure S6. Apoptosis-independent cell death was involved in HA cells treated with C7. HA cells were either untreated or treated with either 20 µM C7, 20 µM Dp44mT or 5nM romidepsin for 48 h. Expressions of Zta , cleaved caspase-3 and -7 were analyzed via Western blot.

Table S1. Relative strength of EBV lytic induction and Fe(III) binding amongst C7 and its analogs.

Compounds	Strength of Fe(III) binding	Effect on EBV lytic reactivation
**C7**	Strong	Strong
**C7-1**	Strong	Strong
**C7-2**	Weak	Weak
**C7-3**	Strong	Strong
**C7-4**	No effect	Weak
**C7-5**	Strong	Strong
**C7-6**	No effect	Weak

The table summarises the strength of lytic induction of C7 and its analogues based on Western blotting results in AGS-BX1, HONE1-EBV and SNU-719 cells. The approximate strength of compounds to Fe(III) was estimated according to the extent of any peak shift in the UV-Vis spectra.

Table S2. Summary of RNA sequencing data of AGS-BX1 cell line with or without treatment by C7.

Sample ID	Number of Raw Reads	Number of Filtered Reads	Number of Reads Mapped in Pair to Human Genome	Number of Reads Mapped in Pair to EBV Genome
**U_8h**	49,302,478	47,098,478	41,904,774	166,024
**U_24h**	52,973,364	50,294,228	45,684,146	142,970
**C7_8h**	58,953,506	55,865,662	38,659,662	1,876,426
**C7_24h**	86,549,530	81,445,950	48,836,622	9,538,502

The table summarises the number of raw reads, reads after quality filtering, reads mapped in pair to human genome (hg19) and reads mapped in pair to EBV genome (NC_007605) after the sequencing run.

© 2018 by the authors. Licensee MDPI, Basel, Switzerland. This article is an open access article distributed under the terms and conditions of the Creative Commons Attribution (CC BY) license (http://creativecommons.org/licenses/by/4.0/).
